# Submandibular Gland Injury With a Ball Bearing Gunshot Wound

**DOI:** 10.7759/cureus.11268

**Published:** 2020-10-30

**Authors:** Cherry Liu, Audric Darian, Laniel Romeus, Santino Cervantes, Tamarah Westmoreland

**Affiliations:** 1 Medicine, University of Central Florida College of Medicine, Orlando, USA; 2 General Surgery, University of Central Florida College of Medicine, Orlando, USA; 3 Radiology, University of Central Florida College of Medicine, Orlando, USA; 4 Pediatric Surgery, University of Central Florida College of Medicine, Orlando, USA; 5 Pediatric Surgery, Nemours Children's Hospital, Orlando, USA

**Keywords:** submandibular, bb gun, submandibular gland trauma, non-powder firearm

## Abstract

Submandibular gland injury is a rare occurrence that has been only documented in case reports. This is due to its protected location under the mandible, and only penetrating injuries to the floor of the mouth or trauma underneath the mandible can reach and damage it. While pediatric injuries due to non-powder firearms are decreasing yearly, 80.8% of the injuries were due to ball bearing (BB) guns. This case report explores the diagnosis and management of a 16-year-old girl who presented with a BB gunshot wound to the submandibular gland. The anatomy, imaging, and surgical management are detailed, and diagnosis guidelines and treatment options are analyzed and explained. This case highlights the importance of understanding the harm that non-powder firearms are capable of causing despite being perceived as toys.

## Introduction

The submandibular glands are one of the three main salivary glands and are located in the posterior portion of the submandibular triangle [1]. This triangle is bordered by the mandibular body (superior), the digastric muscle’s anterior belly (medial), and the digastric muscle’s posterior body (inferior and lateral) [1]. Due to protection from the mandibular body, primarily penetrating trauma or lacerations to the floor of the mouth or trauma underneath the mandible can damage the submandibular gland [2]. These cases are rarely seen and only mentioned in case reports [2]. On the other hand, pediatric injuries from non-powder firearms have averaged 13,486 annually from 1990 to 2016, with ball bearing (BB) guns accounting for 80.8% of the injuries [3]. The study also found that 87.1% of the patients were boys, and the most common injuries were eye injuries, with corneal abrasion as the most common diagnosis [3]. This case presents a 16-year-old girl with a BB gunshot wound to the submandibular gland.

## Case presentation

A 16-year-old girl with no significant past medical history presented to the emergency department with a gunshot wound on the right side of her neck. The patient was shooting a BB gun at a wooden target with a metal base when she heard a metal “cling” sound and felt a pain in the right side of her neck. She stated that initially there was blood loss and applied pressure. When the bandage was removed in the emergency department, there was no active bleeding. Physical examination findings included vital signs within normal ranges and significant right jaw pain with a 2-3 mm circular wound to the right side of her neck with swelling and tenderness. She had no loss of consciousness, difficulty breathing, or difficulty swallowing. She was not in any respiratory distress, and had no stridor or wheezing, with normal effort and breath sounds.

**Figure 1:**
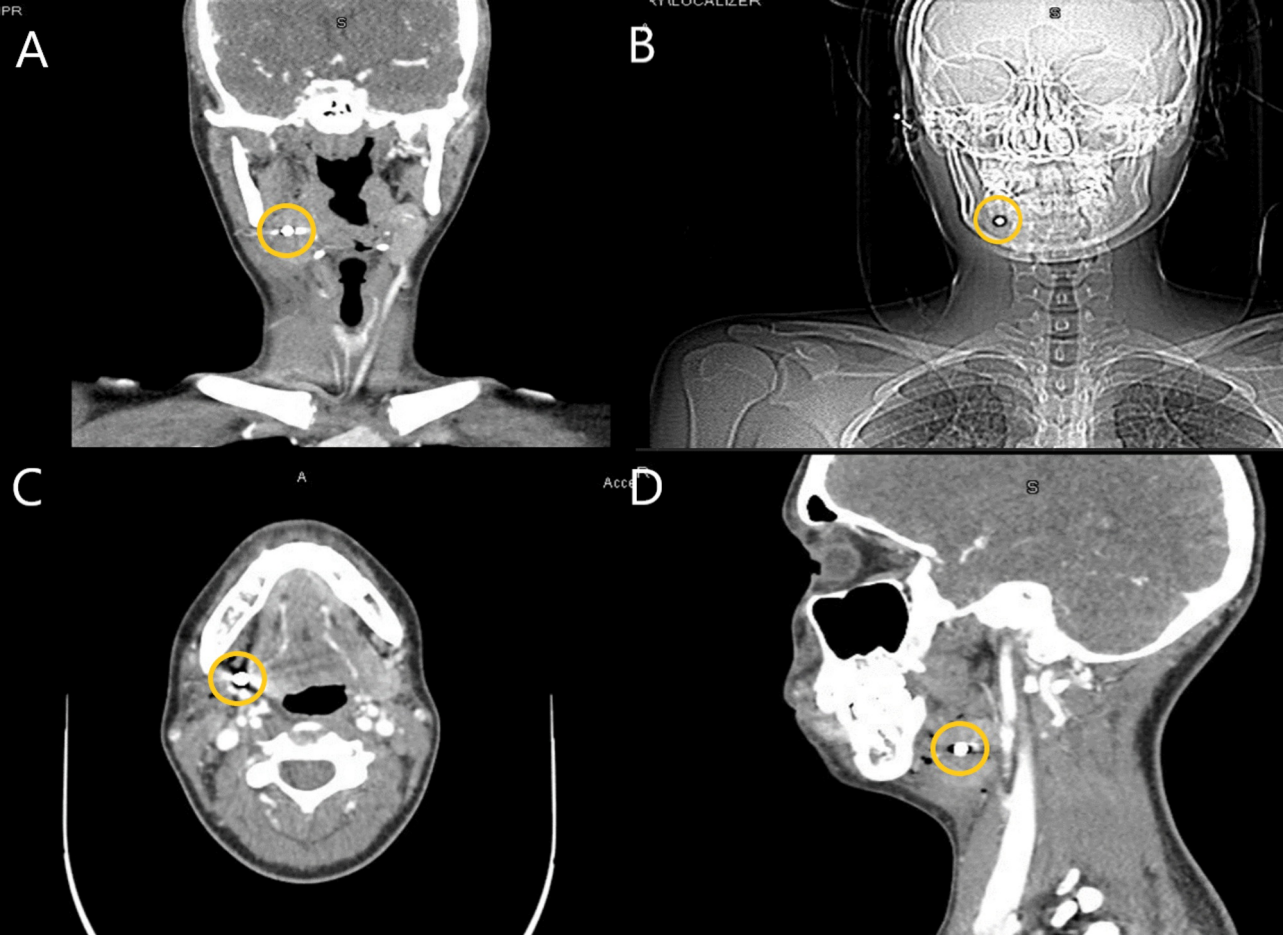
CT scan images from the coronal plane (A), localizer radiograph (B), axial plane (C), and sagittal plane (D) of the BB located in the submandibular gland BB, ball bearing

A soft tissue neck ultrasound revealed a hyperechoic area within the right superficial submandibular region that was 1.2 cm deep to the skin surface. The CT scan of the neck with contrast revealed a 7 mm radiopaque foreign body lodged within the right submandibular gland (Figure 1), There was also evidence of a wound track extending to the right mandible, continuing to the mid-submandibular region. Imaging also revealed extensive soft tissue swelling inferior and lateral to the right-side submandibular gland. The submandibular gland was observed to be swollen, causing mild effacement of the right side airway without extravasation. The wound tract extended from the right submandibular triangle to the right submandibular gland. The chest x-ray was normal.

Because the patient was hemodynamically stable, she was admitted to the hospital overnight for further observation. She remained afebrile and stable throughout her hospital stay, with pain controlled with acetaminophen and morphine. Otolaryngology (ENT) was consulted and their recommendation was outpatient surgery for removal of the submandibular gland foreign body after two weeks to allow the soft tissue swelling to subside. The patient was discharged on oral Augmentin due to the BB remaining within the submandibular gland. The decision to delay the treatment was based on the impressive tissue edema induced by the trauma. Dissection would have been more difficult due to having to dissect through swollen inflamed tissue.

**Figure 2:**
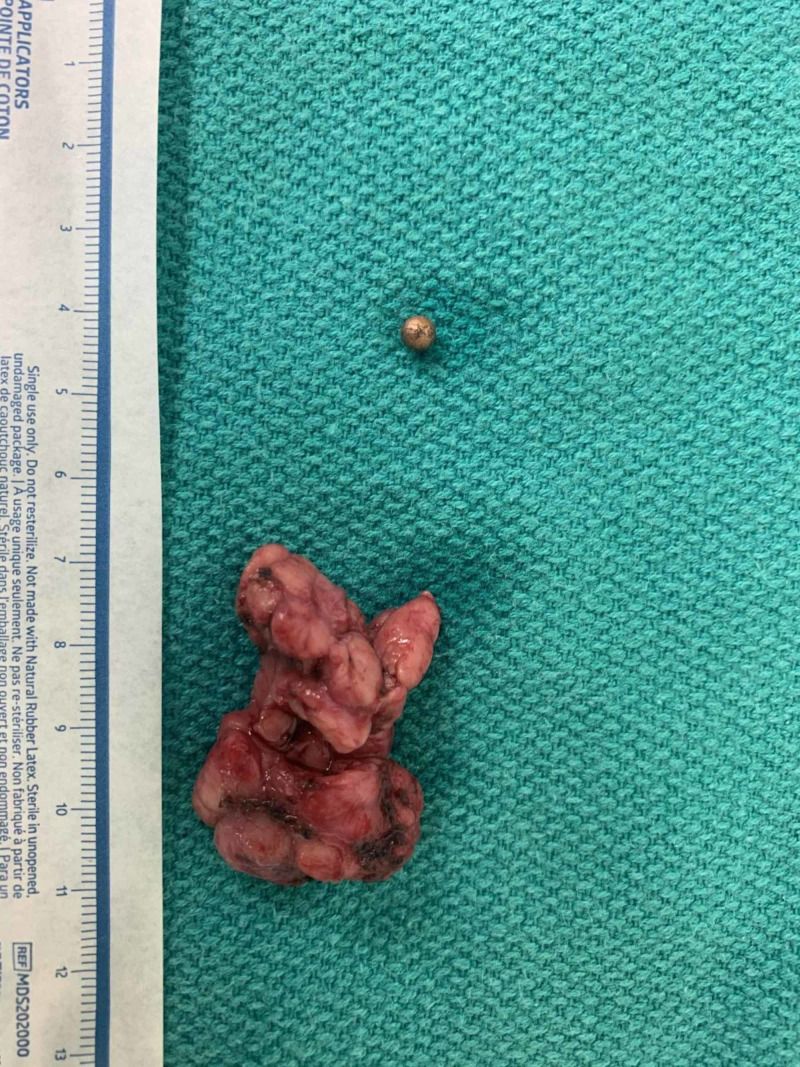
Extracted submandibular gland and BB gun pellet BB, ball bearing

The patient was scheduled for foreign body removal by an ENT specialist 26 days after discharge. She underwent an uncomplicated excision of the submandibular gland and foreign body (Figure 2). Excising the entire gland was necessary since the BB was embedded in the central portion of the gland. There were several attempts to palpate the BB both through the floor of the mouth in hopes that the ENT could extract it through the mouth. Palpation directly on the gland did not locate the BB. The ENT specialist feared if he tried to dissect within the gland without removing it, chronic sialadenitis could occur.

The right submandibular gland was found to have surrounding inflammation and was excised in a routine manner. The BB was located in the center superior portion of the gland. The postoperative course was uncomplicated and the patient was discharged the next day.

## Discussion

Submandibular gland (SMG) injury is typically caused by trauma that fractures the body of the mandible and causes collateral injury posteriorly to the SMG [2]. Due to protection from the mandible superiorly, submandibular gland injury is rarely described, and only occasionally with case studies [2,4]. Injury can also be caused by penetrative trauma from the floor of the mouth or base of the chin [2]. Motor vehicle accidents (MVA) are the most common cause of SMG injury, which usually occurs in conjunction with significant collateral facial trauma [4]. However, any penetrating trauma from the floor of the mouth can cause this, as evident in the case of this patient, in which the pellet ricochet was likely the mechanism of injury. Because current literature details only a few cases of submandibular gland trauma, their etiologies are listed below in Table 1.

**Table 1:** Submandibular gland trauma etiology

Authors	Etiology
Iwai et al. (2018) [5]	Fish bone-induced trauma of the submandibular gland
Harbinson and Page (2010) [4]	Motor vehicle accident with seatbelt compression causing submandibular trauma
Boyd et al. (2002) [6]	Motor vehicle accident with neck hitting the airbag
Tonerini et al. (2002) [7]	Motor vehicle accident
Singh and Shaha (1995) [8]	Traumatic submandibular salivary gland fistula
Roebker et al. (1991) [9]	Motor vehicle accident causing a fractured submandibular gland
de Geus and Maisels (1976) [10]	High-voltage electrical burn of the anterior and left side of patient neck damaging his submandibular gland and requiring removal

As the above table presents, it is rare for a gunshot wound, specifically from a BB gun, to cause submandibular gland injury. Although they can be sold in stores specifically for kids, the U.S. Consumer Product Safety Commission reports about four deaths a year due to BB guns or pellet rifles [11,12]. These BB guns can have muzzle velocities comparable to pistols (750-1450 ft/s) reaching a maximum of 1200 ft/s [13]. A retrospective study utilizing the National Electronic Injury Surveillance System data from 1990 through 2016 found that an estimated 364,133 children were treated in emergency departments across the United States for non-powder firearm related injuries [3]. Another study found that the rates of pediatric eye injury caused by non-powder firearms have increased by over 500% since 2010 [14]. Christoffel and Christoffel concluded that careful supervision of children and adolescents playing with non-powder firearms as well as additional barriers to access to these firearms is imperative to prevent these injuries [15].

Evaluating SMG injury is important because of the anatomy surrounding the gland [1]. Isolated pathology to SMG is seen in cases of neoplasms, autoimmune/inflammatory, and sialadenolithiasis etiologies [2]. In cases of trauma, careful evaluation of collateral damage is done through primary and secondary trauma evaluation [2]. Because MVA is the typical preceding event with facial trauma, consideration of more life-threatening pathology like hematoma compressing trachea should be evaluated in a systematic approach [6]. Regardless of the preceding event trauma evaluation is important in reducing morbidity and mortality from unassessed pathology that requires time-sensitive interventions [6].

Evaluating SMG injury from a trauma inciting event requires a systematic approach [16]. Record of history of injury is always paramount in the diagnosis of any pathology [16]. Imaging should be selected to best suit the data needs of the physician [2]. The primary imaging modality of SMG pathology is best suited when there can be cross-sectional imaging of the complex anatomy surrounding the gland [17]. The most common type of imaging of SMG is a CT scan [17]. Although MRI can provide much more detail, especially in vascular and neoplastic cases, CT imaging is a much more cost-efficient way to manage traumatic injury in a timely fashion [17]. For this patient, in particular, the combination of the situation requiring an acute trauma work-up along with the metallic foreign body made MRI challenging. With imaging of the SMG injury comes the identification of anatomical variance [17]. The submandibular gland sits in a triangle bordered by the mandible and anterior and posterior digastric muscles, which drain to Wharton’s duct [2]. Variance in anatomy must be carefully considered, as understanding patterns of anatomy and relation of lingual nerve, hypoglossal nerve, and submandibular ducts is the most important factor in reducing nerve damage during surgery [18].

Diagnosis and management of SMG injury include surgical management [19]. The most common surgical excision is via the lateral transcervical approach [19]. The biggest disadvantage in the transcervical approach is an injury to local nerves (hypoglossal, facial, and lingual) and scar healing, but transoral may be used in a stable patient with palpable portions of the SMG for resection [19]. The decision for transcervical and transoral should be made by the surgeon on a case-by-case basis. In this case, the operating surgeon decided to complete a transcervical approach because of non-tactile visualization during the oral examination.

Imaging diagnostics depend on if there is associated airway impediment, facial fracture, or nerve palsy, as well as the mechanism of trauma [16]. CT is preferred over MRI because of availability, cost, and time to results [16]. Because the body of the mandible offers protection, injury to the submandibular gland will most likely cause other pathologies identifiable on CT imaging, such as airway obstruction, vascular compromise, and fractures [20].

## Conclusions

Submandibular gland injuries are very rare due to their location underneath the mandibular body offering protection. Thus, they are only described in case reports most commonly due to a motor vehicle accident. In this case, we presented a 16-year-old girl with a submandibular gland injury due to a BB gun accident. While BB guns have been culturally viewed as toys and not dangerous, it is important to provide education and supervision for children operating these guns as they can inflict serious injuries.
